# Molecular Mechanism of Pancreatic Stellate Cells Activation in Chronic Pancreatitis and Pancreatic Cancer

**DOI:** 10.7150/jca.38616

**Published:** 2020-01-14

**Authors:** Guihua Jin, Weilong Hong, Yangyang Guo, Yongheng Bai, Bicheng Chen

**Affiliations:** Key Laboratory of Diagnosis and Treatment of Severe Hepato-Pancreatic Diseases of Zhejiang Province, The First Affiliated Hospital of Wenzhou Medical University, Wenzhou 325000, China

**Keywords:** pancreatic stellate cells, fibrosis, signaling pathway, pancreatic cancer, chronic pancreatitis

## Abstract

Activated pancreatic stellate cells (PSCs) are the main effector cells in the process of fibrosis, a major pathological feature in pancreatic diseases that including chronic pancreatitis and pancreatic cancer. During tumorigenesis, quiescent PSCs change into an active myofibroblast-like phenotype which could create a favorable tumor microenvironment and facilitate cancer progression by increasing proliferation, invasiveness and inducing treatment resistance of pancreatic cancer cells. Many cellular signals are revealed contributing to the activation of PSCs, such as transforming growth factor-β, platelet derived growth factor, mitogen-activated protein kinase (MAPK), Smads, nuclear factor-κB (NF-κB) pathways and so on. Therefore, investigating the role of these factors and signaling pathways in PSCs activation will promote the development of PSCs-specific therapeutic strategies that may provide novel options for pancreatic cancer therapy. In this review, we systematically summarize the current knowledge about PSCs activation-associated stimulating factors and signaling pathways and hope to provide new strategies for the treatment of pancreatic diseases.

## Background

Pancreatic cancer, one of the most malignant cancers in the world, affects over 411,600 people annually, and the 5-year survival rate is still below 5% 1-3. For the vital roles microenvironment played in cancer progression, more and more attention has been paid to it and histological studies showed that stroma cells, especially PSCs, predominated in pancreatic cancer microenvironment.

Pancreatic stellate cells (PSCs) are resident cells of the pancreas and have become a research hotspot in chronic pancreatitis and pancreatic cancer-related fibrosis 4-8. Normally, PSCs are quiescent and regulate extracellular matrix (ECM) production. However, during tumorigenesis, stroma cells and pancreatic cancer cells will secret a variety of stimulating factors (e.g. transforming growth factor-β, TGF-β) to activate PSCs 9. Then, active PSCs can create a suitable microenvironment and facilitate cancer progression by altering four processes in pancreatic cancer models: (I) excessive fibrosis, (II) promoting tumor metastasis, (III) inducting resistance of chemotherapy and radiotherapy and (IV) immune modulation. There have been plenty of evidences that confirmed the importance of PSCs in pancreatic cancer development. Vonlaufen, Hwang, and Gao et al. demonstrated that the cell supernatant of activated PSCs induced cancer cells proliferation and migration mediated by PDGF and SDF-1/CXCR 4, 8, 10. It also found that PSCs significantly promoted the growth and metastasis of cancer cells by co-culturing PSCs with pancreatic cancer cells 11, 12. Apte et al. discovered that PSCs can induce tumor-promoting paracrine effects 13. And Liu et al. found activated PSCs decreased the apoptosis of pancreatic cancer cells induced by gemcitabine 14. So, it is crucial to find out the active partners and how they work. Therefore, the purpose of this review is to summarize the knowledge about the molecular basis involved in the activation of PSCs in chronic pancreatitis and pancreatic cancer.

## Stimulators and PSCs activation

In recent years, increasing evidence shows that many extracellular signals exert a significant effect on fibrosis development during chronic pancreatitis and pancreatic cancer. These stimulating factors are able to work in the activation of PSCs and may be divided into five groups: (I) cytokines/transcription factors, (II) non-coding RNAs, (III) oxidative stress related factors, (IV) hyperglycemia and (V) ion channels and calcium signaling.

### Cytokines/Transcription factors

#### TGF-β

TGF-β is one of the most potent regulatory cytokines of the fibrotic response. It has been observed that increased expression of TGF-β in the injured acinar cells which were adjacent to areas of fibrosis 15. TGF-β1 could activate MAPKs signal pathway and induce an enhanced mRNA expression level of JNK1 and ERK1, which then leading PSCs to differentiate into myofibroblasts that could secret a variety of ECM, such as type I collagen and fibronectin 16-18. Further studies have demonstrated that PSCs synthesize TGF-β1 itself, which suggesting the possible existence of autocrine loops that may contribute to the continuous activation of PSCs after an initial exogenous signal 19. Vogelmann et al. found that on the 14th day after birth of TGF-β1 overexpressing transgenic mice that have the morphological features of chronic pancreatitis, the fibrous tissue was mainly composed of type I and III collagens 16. And on 70th day, the elevated deposition of laminin amounted to the over-synthesis of ECM. PSCs were the principal cellular source of type I collagen in pancreatic fibrosis in both humans and in an experimental animal model. Taking advantage of trans retinoic acid, that could weaken the biological activity of TGF-β, hampered the active myofibroblast PSCs phenotype, which is strongly associated with tumor growth and metastasis 20, 21. So, this indicating that TGF-β1 was possibly responsible for pancreatic fibrosis through the activation of PSCs.

In addition, activin A, a member of the TGF-β superfamily, was detected to work with TGF-β1 and increased each other's secretion and mRNA expression in PSCs 22-24. And taking advantage of endogenous binding protein for them could reverse PSCs activation (e.g. follistatin) 25. However, it still needs fuither study to find out the details and tell the differences between TGF-β1 and activin A in PSCs activation.

#### Connective tissue growth factor (CTGF)

CTGF is the member of the CNN family proteins. Studies have shown that CTGF, similar to TGF-β, is an autocrine regulator of PSCs 26, 27. It has been known that CTGF was produced at the injury site by acinar cells or fibroblastic cells, and these fibroblastic cells, produced the highest level of CTGF, were likely include a population of activated PSCs 28, 29. Besides, it can also affect PSCs activation by TGF-β 30, 31. Abreu et al. found that the presence of CTGF would increase the cross-linking between TGF-β1 and all of its receptor binding proteins 31, 32. Neutralizing antibody of CTGF inhibited the fibrotic effects of TGF-β1, suggesting that CTGF and TGF-β1 may have a synergistic effect. Moreover, TGF-β1 could also activate the CTGF promoter in a short term 30. And down-regulation of CTGF/CCN2 expression by small interfering RNA caused a reduction of PSC proliferation 27.

#### Interleukin-10 (IL-10)

IL-10, another important autocrine regulator of PSCs, is a potent anti-inflammatory and anti-fibrotic cytokine produced by Th2 cells 33. Endogenous IL-10 might directly limit the atrophy and fibrosis of pancreatic gland by downregulating procollagen I and enhancing collagenase gene expression^34^-^37^. Meanwhile, it cuts down the release and expression of TGF-β in the pancreatic acinar and stromal cells 38-40. Demols et al. revealed that IL-10 deficient chronic pancreatitis mice had severer damage and fibrosis of pancreatic tissue, and the level of plasma TGF-β1 and the number of activated PSCs were all significantly raised as well 38. So, IL-10 is probably an effective target to inhibit the pancreatic fibrosis.

#### Platelet derived growth factor (PDGF)

PDGF is mainly synthesized by inflammatory cells and is a crucial stimulator in activating PSCs and forming of ECM 41, 42. PDGF family consists of four different polypeptide chains encoded by four different genes: four homodimers of A, B, C or D subunits and one heterodimer AB. And they act via two receptor tyrosine kinases, PDGF receptors (PDGFR) α and β 43. It has been shown that the expression of PDGFR-β in PSCs correlates with the prognosis of patients with pancreatic cancer 44. However, detailed molecular mechanisms remain unclear. *In vitro*, activated PSCs express PDGFR-α and PDGFR-β 15, 45, 46. PDGF-BB induces the phosphorylation of PDGFR-β, and subsequently activates phosphatidylinositol 3 kinase (PI3K) and extracellular regulated protein kinase (ERK) pathways 46. ERK pathway regulates the proliferation and migration of PSCs, and blocking the ERK pathway could completely suppress the proliferation and almost 50% cell migration of PSCs 46, 47. However, inhibition of PI3K pathway did not affect PDGF-BB induced proliferation 46. Thus, PDGF is the most potent mitogen for PSCs proliferation, and stimulates the synthesis of collagen and fibronectin as well 46, 48.

#### Hypoxia inducible factor-1α (HIF-1)

Hypoxia contributes to the development of pancreatic diseases. Recent studies have shown that a hypoxic environment exists not only in cancer cells, but also in surrounding PSCs 49. The response of the PSCs to hypoxia is achieved by HIF-1 which is a heterodimeric protein containing both α and β subunits. HIF-1β expresses in normoxia, while HIF-1α only accumulates in the state of hypoxia. Subsequently, HIF-1α translocates to the nucleus and participates in the transcription of vascular endothelial growth factor and other pro-fibrotic genes in PSCs 50. Activated HIF-1α could also mediate the upregulation hepatoma-derived growth factor gene which finally contributes to the antiapoptosis of PSCs and consequently leads to the synthesis and deposition of ECM proteins 51.

### Non-coding RNAs

Recently, numerous families of non-coding RNAs, especially microRNAs (miRNAs), have attracted the attention of researchers for their dramatically altered expression profiles during the PSCs activation 52.

Some miRNAs expressions are beneficial to PSCs activation. Masamune et al. found that pancreatic cancer cell-derived exosomes could increase the activity of PSCs and also induce their fibrosis-related gene expression by miR-1246 and miR-1290 53. Connective tissue growth factor (CCN2) and miR-21 are components of PSCs-derived exosomes which are significantly up-regulated in activated PSCs. The expression level of CCN2 drives miR-21 induction which acts via positive feedback loop to potentiate CCN2 production, and finally amplifying collagen production in the cells. It's revealed that exosomes, containing miR-21 and CCN2 transcripts, enabled their uptake by other PSCs and influenced the phenotypes of PSCs 54, 55. And the transfection of active anti-miRNA-199a oligonucleotide into human PSCs could lead to the inhibition of PSCs activation and differentiation into cancer-associated fibroblasts 56, 57.

However, some miRNAs have the opposite effect. Asama et al. found that the miRNA let-7d-5p inhibited the activation of human PSCs by targeting thrombospondin 1 and downstream TGF-β pathway 58, 59. Wang et al. reported that the inhibition of syntaxin-12 lncRNA, which making miR-148a upregulated and Smad5 decreased, ultimately resulting in the suppression of PSC activation 60. Liu et al. had a similar discovery that lncRNA myocardial infarction-associated transcript contributed to PSCs activation through suppressing miR-216a-3p-mediated COX-2, which finally leading to pancreatic fibrosis 61. Same studies also uncover the inhibitory effect of miR-200a in PSCs activation 62.

Since more and more studies have found the roles of miRNAs in PSC activation, further studies are needed to investigate where are the exact origins of these miRNAs, PSCs or cancer cells, and how they really work.

### Oxidative stress related factors

According to experimental chronic pancreatitis and clinical observations, oxidative stress exerts an enormous function on PSCs activation and pancreatic fibrosis. Exogenous H_2_O_2_, a traditional cellular reactive oxygen species (ROS) inducer, promoted PSCs α-smooth muscle actin (α-SMA) production and migration 55. Zeki et al. revealed that the level of oxygen free radicals elevated with the change of pancreatic fibrosis in wBN/Kob rats and reduced the activity of SOD 63. Applying DA-9601, an anti-oxidant chemical substance, the inflammation and myeloperoxidase activity of chronic pancreatitis mice were both decreased, and the expression of α-SMA and type I collagen in isolated PSCs also reduced 64. In recent years, coenzyme Q10, a powerful antioxidant, was also found could ameliorate pancreatic fibrosis via reducing intracellular levels of ROS in PSCs 65, 66.

Ethanol and smoking are now recognized as important risk factors for chronic pancreatitis. It's largely because both pancreatic acinar cells and PSCs metabolize ethanol to generate oxidative stress that promote PSCs activation and lipid peroxidation 67, 68. Meanwhile, ethanol and its metabolites also upregulated the activity of MAPK pathway and the expression of α-SMA in PSCs 69. SB203580, which was used to block p38 kinase, inhibited the spontaneous activation of PSCs, suggesting that it was p38 MAPK pathway which took effects on PSCs activation. As for smoke, previously studies reported that nicotine and nicotine-derived nitrosamine ketone and cigarette smoke extracts induced nAChR (isoforms α3, α7, β, ε) expression in the cells 70, and increased production of ROS in PSCs 71. Aryl hydrocarbon receptor ligands in cigarette smoke could upregulate IL-22 in PSCs which induced the expression of the extracellular matrix genes fibronectin 1 and collagen type I α1 chain 72. However, the mechanisms mediating the effects of smoking on PSC activation are still largely unknown.

Ductal hypertension has been believed to be a major contributor of biliary pancreatitis. Asaumi et al. found that pressure could induce PSCs activation and increase ROS level in activated PSCs 73. And Watanabe et al. found that enhanced pressure mainly induced the 5-bromodeoxyuridine incorporation and α-SMA expression 74. In addition, intracellular pressure rapidly elevated the phosphorylation of ERK and p38 MAPK and promoted the secretion of TGF-β1 and collagens. The antagonists of mitogen-activated extracellular signal-regulated kinase (MEK) and p38 MAPK inhibited stress-induced α-SMA expression and cell proliferation. Thus, increasing pressure of pancreatic tissue may accelerate the progress of fibrosis in chronic pancreatitis by activating PSCs.

Therefore, the above studies suggest that oxidative stress related factors play a role in the occurrence of pancreatic injury and fibrosis, to some extent, by activating PSCs.

### Hyperglycemia

Observations from previous studies have showed that high glucose resulted in more α-SMA and ECM proteins expression 75. Subsequently, researchers indicated that high glucose may activate PSCs through p38 MAPK pathway and finally resulted in increased ECM production 76, 77. And chronic hyperglycemia could not only activate PSCs, but also promote the interaction of PSCs and pancreatic cancer cells 78. Increasing attentions still need to be directed towards the role of hyperglycemia in PSCs activation.

### Ion channels and calcium signaling

Besides, growing evidence has revealed the crucial role of calcium signaling and ion channels played in PSCs stimulated by the above factors 79-86. Schwab et al. found that several members of the transient receptor potential (TRP) family contributed to PSCs activation. In pressure-induced PSCs activation, transient receptor potential canonical 1 (TRPC1)-mediated calcium influx was increased 81. However, knockout of TRPC1 led not only to attenuated phenotype and cytokine production, but also a reduced Ca^2+^ influx in PSCs. As for TRPC3, PDGF could stimulate migration of PSCs in a K_Ca_3.1 channel dependent manner, and knockdown of TRPC3 channels largely abolished this impact on PSC migration for their provision with Ca^2+^ required for channel activation 80. Similar as the function of TRPC1 channels, hypoxia could also induce PSCs activation in a TRPC6-dependent manner 79. In addition, Yule and Petersen et al. proposed a novel idea of PSCs activation. They found PSCs in their normal microenvironment are far from quiescent, they can generate substantial cytosolic Ca^2+^ signals in response to the stimulation of the blood pressure-lowering nona-peptide bradykinin and some other substances 83, 84. However, more efforts still need to be taken to work out the actual state of PSCs activation.

## Signaling pathways and PSCs activation

**Figure 2:**
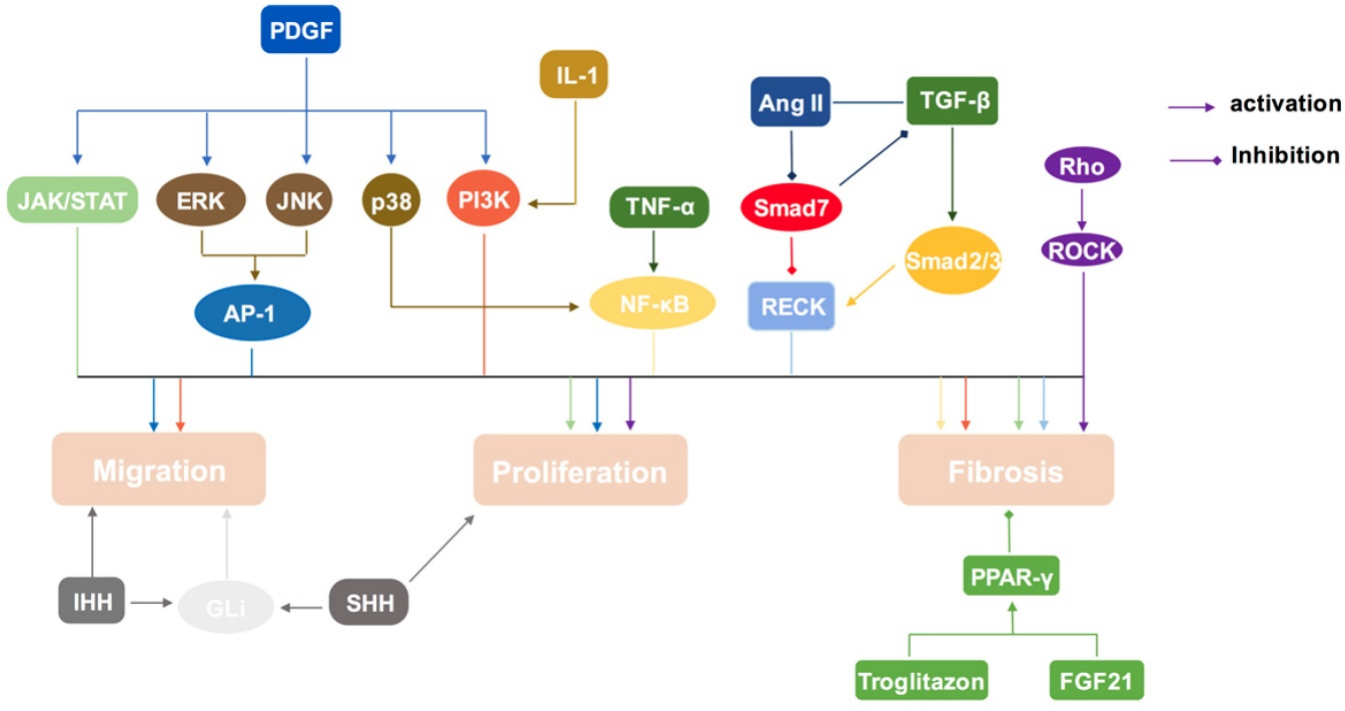
Signal transduction pathways in PSCs activation. PSCs can be activated by MAPK, Rho/ROCK, NF-κB, PI3K/AKT, JAK/STAT, and sonic hedgehog-Gli1 pathway. Smads pathway play a dual role in this process. And PPAR-γ pathway will inhibit the activation of PSCs.

Accumulating studies are aiming at seeking methods to inhibit or reverse the activation of PSCs, which finally amounts to preventing or delaying the fibrosis process in pancreatic diseases. PSCs activation involves in several important signal transduction pathways (Table 1, Figure 2). Thus, deeply exploring the role of these pathways is of great significance in the treatment of chronic pancreatitis and pancreatic cancer.

### MAPKs

Previous research reported that MAPK signaling pathway was involved in the early phase of acute pancreatitis 87. In respond to extracellular stimuli, MAPKs take effects on many cellular events, such as proliferation, apoptosis and survival, and can upregulate the expression of inflammatory cytokines in the pancreas 88, 89. The central members of the MAPKs (ERK, JNK, and p38 MAPK) could transduce signals that are generated by cytokines, growth factors, and intracellular stress. ERK, JNK, and p38 MAPK have been reported increased in mice chronic pancreatitis model, and PSCs were the source of producing MAPKs 17.

Cascade performance in ERK signaling pathway is starting from stimulating receptor tyrosine kinases (RTKs), and then activating of Raf and RasGTP enzyme. ERK, which is activated by Raf, translocates to the nucleus to regulate transcription factors, such as activator protein-1 (AP-1) 90. AP-1 is a transcription factor which can be phosphorylated by the mitogen-activated protein kinase (MAPK) family members 91, 92. Owing to MAPK pathway is involved in the PSCs activation, it suggests that AP-1 may also refer to activate PSCs 46, 93. Schwer et al. reported that curcumin induced the expression of oxygenase-1 (HO-1) gene, thereby suppressing the activation of ERK 1/2 and subsequently inhibiting the proliferation of PSCs 94. Adding ERK inhibitors to PSCs, the expressions of CX3CR1 and SMA in PSCs decrease dramatically, suggesting that ERK 1/2 may participate in the process of fibrosis by regulating chronic pancreatitis-related cytokines 95. Above all, studies have shown that ERK pathway acts on the migration, activation and matrix synthesis of PSCs 96.

C-Jun amino terminal kinase (JNK) is phosphorylated by MAP3Ks (such as ASK1, MEKK1, MLK3) and MAP2Ks (such as MKK4, MKK7) after activated by cytokines, pressure and other factors 97. Phosphorylated JNK binds to activating transcription factor 2 (ATF2) through the amino terminal domain of c-Jun to form a dimer that enhances the transcriptional activity of AP-1. Fitzner et al. found that the presence of JunD in AP-1 complexes was typical for activated PSCs, while the portion of JunB-containing AP-1 complexes decreased during the process of activating PSCs, along with the overall decrease of AP-1 DNA binding activity as well 98. And in freshly isolated PSCs, the JNK inhibitor curbs IL-1β-induced activities of JNK and AP-1, as well as the PDGF-mediated activation of PSCs 99. Studies of knockout mice have shown that MAPK phosphatase (MKP) plays a negative regulatory role in JNK pathway, and the use of reactive oxygen species (ROS) to inhibit MKP activity can prolong the activation of JNK 100. Moreover, JNK and ERK were believed respond directly to TGF-β1 and PDGF, which are considered as the most important factors of PSCs proliferation and ECM deposition 17, 46.

p38, a kind of stress-activated protease (SAPK), is activated by a variety of proinflammatory-related factors, and takes an effect on apoptosis, transcriptional regulation, cytokine production and cytoskeleton recognition 101. Freshly isolated mouse PSCs were treated with SB203580, a specific inhibitor of p38 MAPK, and the levels of α-SMA and type I collagen in PSCs were significantly reduced 102. Seven days Later, the activation of PSCs was not observed yet, indicating that activated p38 MAPK may participate in PSCs activation. Apte et al. reported that antagonizing p38 MAPK other than ERK or JNK pathways, inhibited PSCs activation and α-SMA expression, suggesting that p38 probably plays a major role in acetaldehyde-mediated PSCs activation 103. Although ethanol and acetaldehyde-induced PSCs activation could upregulate the ERK 1/2 and p38 expression, only the inhibition of the p38 MAPK pathway decreased α-SMA expression and the activation of PSCs 69, 103.

Nevertheless, the specific mechanisms need further exploration.

### Ras homolog gene family/rho-associated protein kinase (Rho/ROCK)

Studies have shown that small GTP-binding protein Rho and its downstream effector ROCK act on actin cytoskeleton, stress fibers and cell morphology 104. It is reported that the expression of stress fibers on the surface of activated PSCs increases before the whole cells are filled with stress fibers. Y-27632 and HA-1077, specific inhibitors for ROCK, prevent spontaneous activation of PSCs 73, 105. The stress fibers decompose gradually after Y-27632 acting on activated PSCs, indicating that PSCs activation is related to Rho/ROCK pathway. Additionally, Y-27632 reduces the expression of α-SMA, one of the markers of PSCs activation, hinders the proliferation and chemotaxis of PSCs mediated by PDGF, and prevents the production of collagens. Thus, it is implicating that Rho/ROCK pathway not only induces PSCs active, but also promotes its proliferation and chemotaxis.

### NF-κB

The pleiotropic transcription factor NF-κB is composed of homologous or heterodimer of the Rel protein family members (p65, p50, p52, c-Rel, and RelB). In mammalian cells, the synthesis of classic NF-κB complex, constituted by p65/p50 heterodimer, could be triggered by cytokines, mitogen, ultraviolet and other stimulating factors, such as tumor necrosis factor-α (TNF-α) and IL-1 106. TNF-α and IL-1 induce activated PSCs to express IL-6, IL-8 and monocyte chemoattractant protein-1 (MCP-1) through the AP-1, which are target genes of NF-κB, showing that NF-κB activation may promote pancreatic fibrosis through PSCs 93, 107, 108. Recent studies have shown that Bay11-7082, the specific blocker of NF-κB, hamper NF-κB pathway by clearing up MCP-1, IL-8 or nitric oxide synthase (NOS) that are induced by Toll-like receptor (TLR) and galactin-1, demonstrating that TLR and galactin-1 may promote PSCs activation and fibrosis through NF-κB pathway 109. Further study found that pancreatic ductal adenocarcinoma (PDAC)-derived galactin-3 activated PSCs and promoted PSCs secretion of proinflammatory factors (IL8, CCL2, and CXCL1) through ITGB1/ILK/NF-κB signaling cascade 110.

### Peroxisome proliferator activated receptor-γ (PPAR-γ)

PPAR-γ, a nuclear hormone receptor, is involved in adipocyte differentiation, proliferation, immune response, and insulin secretion in adipose tissue and immune system 111. The researchers reveal that the expression of PPAR-γ is negatively correlated with PSCs proliferation, and the ligand of PPAR-γ, Troglitazone, can reduce α-SMA expression and PSCs proliferation by PPAR-γ signaling 112, making the activated PSCs transform to the stationary state 113. Consequently, it alleviates the process of pancreatic inflammation and fibrosis, and finally meliorated the development of chronic pancreatitis in animal models 114-117. Johnson et al. reported that fibroblast growth factor 21 (FGF21) is an important factor involved in lipid metabolism that regulates the activity of PSCs through PPAR-γ pathway 118.

### PI3K-Serine/threonine kinase (AKT)

The PI3K family is a class of kinases that specifically catalyses the phosphorylation of PI3-hydroxyl groups to produce inositol ester material that acts as a second messenger. PI3K attracts Akt (called protein kinase B) into the cell membrane during phosphorylation 119. PI3K/Akt pathway exerts an effect on cell proliferation, anti-apoptotic, migration, transmembrane translocation and cell carcinogenesis 120. It's known that IL-1β, TNF-α and IFN-γ regulate the activity of PSCs to express IL-32α that triggers fibrosis, and the inhibitor of PI3K downregulates IL-32α expression in response to the stimuli 121. Nishida et al. reported that PDGF regulated the migration of PSCs by activating the PI3K/Akt pathway 121, 122. What's more, this pathway had a cross effect with the MAPK pathway in aldehydes-induced PSCs activation 103. Blockade of the PI3K/Akt pathway with carbon monoxide releasing molecule-2 (CORM-2) led to down-regulation of cyclin D1 or E and arrestment in G_0_/G_1_, and finally inhibited the activation of PSCs 123, 124. However, recently, Cui et al have found that activating the PI3K/Akt/mTOR pathway could inhibit autophagy and further suppress PSCs activation 125-127. They also suggested that TGF-β1 might be regulated by PI3K/Akt pathway, but the significance of this crosstalk still required further study 128-130.

### Janus-activated kinase (JAK)/signal transducer and activator of transcription (STAT)

JAK is a group of receptor-deficient tyrosine kinases activated by phosphorylating transcription factor STAT 131. The STAT proteins are present in the cytoplasm as precursor proteins, and the tyrosine residue is phosphorylated and translocated to the nucleus to bind to the specific DNA. It's known that PDGF promotes the proliferation of PSCs through the JAK/STAT pathway 48. PDGF-mediated activation of STAT1 and STAT3 was blocked by the inhibitors of Src and JAK2, resulting in the reduction of fibrosis 132. Therefore, it's speculated that the activation of the Src-dependent JAK/STAT pathway is involved in PSCs activation and PDGF-mediated fibrosis. In pancreatic acinar cells, bombesin activates the JAK/STAT pathway via nitrogen oxides (NOX). In PSCs, ROS may also contribute to the activation of NF-κB and JAK/STAT pathways in the development of acute pancreatitis 133.

### Smads

The Smads family, a group of small molecules that regulate intracellular signals, has been found to have at least nine members that divided into three groups, including pathway restriction proteins 134, co-mediated type proteins 135, and inhibitory proteins 136. It's reported that Smads are functionally dynamic in PSCs 137. Firstly, TGF-β promotes Smad2, 3 and 4 to form a polymer and subsequently enters the nucleus to regulate the transcription of the target gene. TGF-β promotes the fibrosis of pancreas through Smad2 or Smad3 pathway, however, inhibits the expression of Smad7 96. Moreover, angiotensin II inhibits the expression of Smad7 mRNA through the PKC pathway, and promotes the TGF-β-mediated proliferation of PSCs 138. Smad4, a key downstream protein for TGF-β/Smad pathway, regulates plasminogen activator inhibitor 1 (PAI-1) and participates in the production of ECM. Smad3/4 binds to two adjacent Smad-binding sites in TGF-β reaction region and initiates the transcription of PAI-1 to increase the precipitation of ECM afterward. The interaction of Smad7 with TGF-β receptor (TβR) blocks TGF-β signal translocation to the nucleus and the phosphorylation of Smad2/3, thereby preventing PSCs activation. After the treatment of TGF-β, decreased Smad7 and raised Smad3 expression, and gradually activated PSCs are all in a concentration-dependent manner. Smad7 also hampers the overexpression of tissue inhibitor of metalloproteinases (TIMPs) that mediated by TGF-β, reducing the formation of collagen fibers and deposition of ECM 138.

The reversion-inducing-cysteine-rich protein with kazal motifs (RECK), a kind of PSC membrane anchor proteins, regulates the activity of cell surface matrix metalloproteinases (MMPs). Pancreatic acute phase protein and chronic time-dependent protein can downregulate RECK protein expression, thereby affecting the activity of MMPs and the metabolism of ECM 139. The expression of RECK is regulated by Smads system, and Smad7 overexpression or decreased Smad3 expression may result in reduced RECK 140. Bone morphogenetic protein 2 (BMP2), a member of the TGF-β family, is able to bind the receptor to phosphorylate Smad1/5/8. Gao et al. reported that the level of BMP was high in pancreatic tissue induced by bombesin, and the deletion of BMP2 increased fibrosis in chronic pancreatitis rats 141. Consequently, it is expected that the anti-fibrosis effect of BMPR2-Smad1/5/8 signaling pathway works through inhibiting TGF-β/Smad2 and p38 pathway in pancreas.

### Hedgehog

Sonic hedgehog (Shh) and indian hedgehog (Ihh) are the major members of the Hedgehog family. Shinozaki et al. found that activated PSCs expressed patched-1 (Ptch1) and smoothened (Smo) which were both important components of the Hedgehog receptor system 142. Ihh enhances the migration of PSCs in terms of chemotaxis and chemical activation, and increases the expression of MMP1 which degrades the basement membrane to promote cell movement. TIMP2 can attenuate the migration of PSCs caused by Ihh stimulation. Most of the hedgehog intercellular signal transductions are regulated by the transcription factor Gli1. And Ihh induces the accumulation of Gli1 in the nucleus of PSCs, suggesting that Ihh may activate Gli1-dependent signaling pathways. Shh which produced by pancreatic cancer cells is able to activate quiescent PSCs, induce them to express Gli1 and further promote cell migration 143-145. Accordingly, hedgehog signaling is an indispensable pathway in the activation of PSCs and the production of ECM during fibrosis in chronic pancreatitis and pancreatic cancer 146.

## Conclusion

In recent years, increasing attention has been directed to the role of activated PSCs in pancreatic diseases, especially chronic pancreatitis and pancreatic cancer. Quiescent PSCs are involved in maintenance of pancreatic tissue architecture and maintain ECM turnover 147. However, activated PSCs exhibit deranged ECM turnover and contribute to inflammatory microenvironment. Although a few prominent publications described that the presence of stroma rich in activated PSCs greatly restrained tumor progression 148, 149, it is still an exciting challenge to understand the detailed mechanisms that govern the process of PSCs phenotype transition in the distinct phases of pancreatic inflammation and tumorigenesis. As described above, we have some knowledge about PSCs activation. Nevertheless, still much has to be learned. Making the complex networks in PSCs activation clear will help us to explore deeply about the molecular mechanism of pancreatic fibrosis and probably help to aid the development of novel therapeutic strategies against pancreatic cancer.

## Figures and Tables

**Figure 1:**
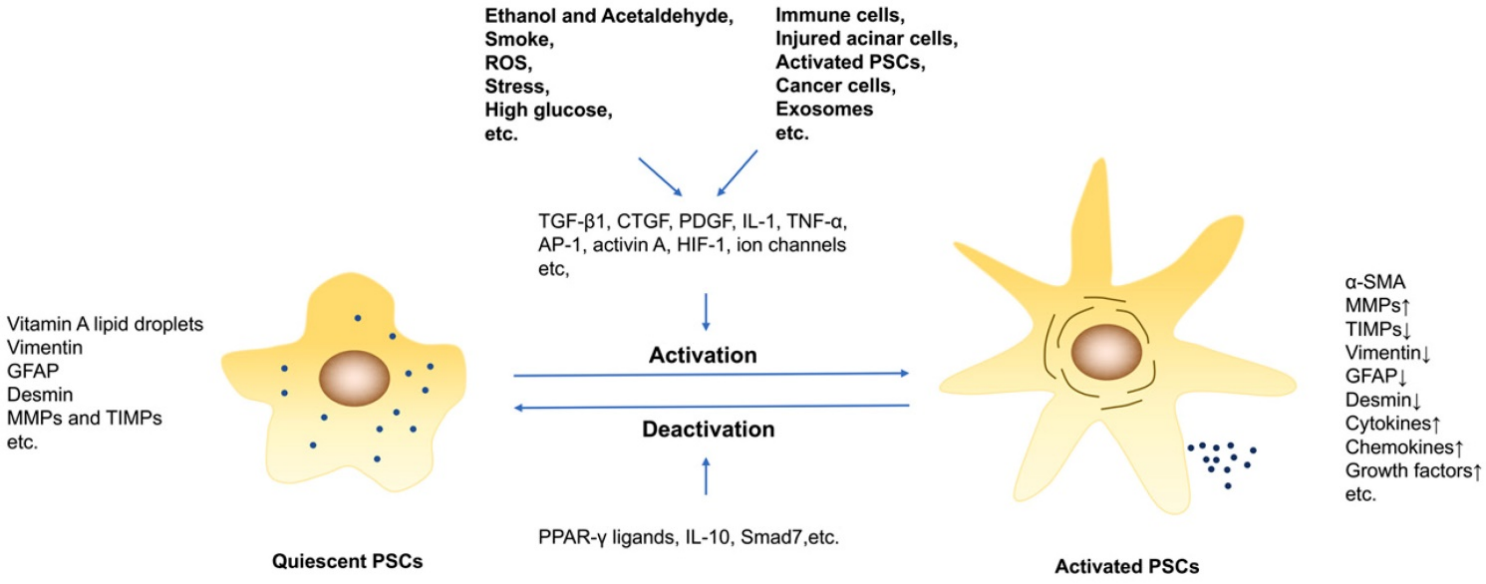
The activation and deactivation of PSCs. PSCs are activated by profibrogenic mediators, such as ethanol, high glucose and cytokines. And persisting of PSCs activation under pathological conditions results in pancreatic fibrosis.

**Table 1:** The signal transduction pathways in PSCs

Pathway	Function	Reference
MAPK(ERK)	Migration, Proliferation	90, 94-96
MAPK(JNK)	Migration, Proliferation, Cytokine production	17, 46, 97, 99, 100
MAPK(p38)	Fibrosis, α-SMA expression	69, 102, 103
Rho/ROCK	Fibrosis, Proliferation, Chemotaxis	73, 104, 105
NF-κB	Fibrosis	106, 108-110
PPAR-γ	Anti-fibrosis, Maintain the quiescence	111-118
PI3K/Akt	Migration, Fibrosis	103, 119-123
JAK/STAT	Proliferation, Fibrosis	48, 131-133
Smads	Dual role of fibrosis	96, 137-141
Hedgehog	Migration, Proliferation	142-146

## Abbreviations

AP-1: activator protein-1
Ang II: Angiotensin II
α-SMA: α-smooth muscle actin
CTGF: connective tissue growth factor
ECM: extracellular matrix
ERK: extracellular regulated protein kinase
FGF21: fibroblast growth factor 21
GFAP: glial fibrillary acidic protein
HIF-1: hypoxia-inducible factor
IHH: indian hedgehog
IL: interleukin
JNK: c-Jun N-terminal kinase
JAK/STAT: janus-activated kinase/signal transducer and activator of transcription
MAPK: mitogen-activated protein kinase
NF-κB: nuclear factor-κB
PI3K/Akt: phosphatidylinositol 3 kinase-serine/threonine kinase
PSCs: pancreatic stellate cells
PPAR-γ: peroxisome proliferator activated receptor-γ
MMPs: matrix metalloproteinases
PDGF: platelet derived growth factor
RECK: the reversion-inducing-cysteine-rich protein with kazal motifs
Rho/ROCK: ras homologue gene family/rho-associated protein kinase
RTKs: receptor tyrosine kinases
SHH: sonic hedgehog
TGF-β1: transforming growth factor-β1
TIMPs: tissue inhibitor of metalloproteinases
TNF: tumor necrosis factor
TRP: transient receptor potential
TRPC1: transient receptor potential canonical 1
